# A second monoclinic polymorph of *N*-[bis­(morpholin-4-yl)phosphino­yl]-4-fluoro­benzamide with the *P*2_1_/*n* space group

**DOI:** 10.1107/S1600536812031418

**Published:** 2012-07-14

**Authors:** Atekeh Tarahhomi, Mehrdad Pourayoubi, Mojtaba Keikha, Arnold L. Rheingold, James A. Golen

**Affiliations:** a Department of Chemistry, Ferdowsi University of Mashhad, Mashhad, Iran; b Department of Chemistry, University of California, San Diego, 9500 Gilman Drive, La Jolla, CA 92093, USA

## Abstract

A second monoclinic polymorph of the title mol­ecule, C_15_H_21_FN_3_O_4_P, is reported in the space group *P*2_1_/*n* and compared to the previously reported *C*2/*c* space group [Gholivand *et al.* (2006 ▶). *Polyhedron*, **25**, 711–721]. The asymmetric unit of the title compound consists of two independent mol­ecules. The P atoms adopt a distorted tetra­hedral environment. In the C(O)NHP(O) fragment, the P=O and the N—H groups are in a *syn* conformation with respect to each other and in the crystal, inter­molecular N—H⋯O=P hydrogen bonds form dimeric aggregates.

## Related literature

For the monoclinic polymorph of the title mol­ecule, in a *C*2/*c* space group, for bond lengths and angles and for preparation of the starting compound 4-F—C_6_H_4_C(O)NHP(O)Cl_2_, see: Gholivand *et al.* (2006 ▶). For related phospho­ramidates, see: Pourayoubi, Nečas & Negari (2012 ▶); Pourayoubi, Tarahhomi *et al.* (2012 ▶).
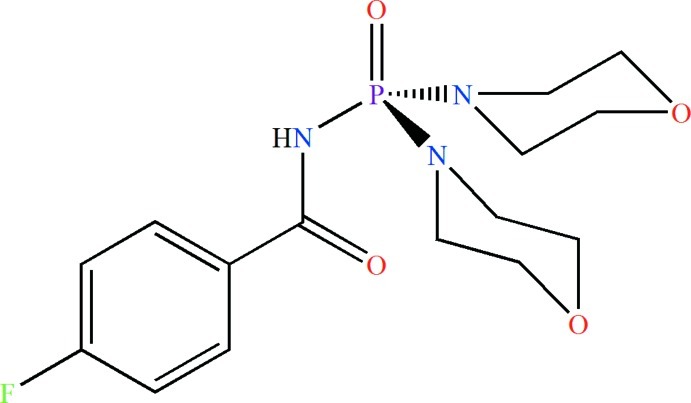


## Experimental

### 

#### Crystal data

C_15_H_21_FN_3_O_4_P
*M* *_r_* = 357.32
Monoclinic, 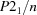

*a* = 15.6093 (6) Å
*b* = 10.7114 (4) Å
*c* = 21.0045 (9) Å
β = 106.896 (2)°
*V* = 3360.3 (2) Å^3^
*Z* = 8
Mo *K*α radiation
μ = 0.20 mm^−1^
*T* = 100 K
0.18 × 0.15 × 0.10 mm

#### Data collection

Bruker APEXII CCD diffractometer
Absorption correction: multi-scan (*SADABS*; Sheldrick, 2004 ▶) *T*_min_ = 0.965, *T*_max_ = 0.980
25230 measured reflections
6912 independent reflections
5355 reflections with *I* > 2σ(*I*)
*R*_int_ = 0.037

#### Refinement

*R*[*F* ^2^ > 2σ(*F* ^2^)] = 0.056
*wR*(*F* ^2^) = 0.160
*S* = 1.03
6912 reflections
439 parameters
2 restraints
H atoms treated by a mixture of independent and constrained refinement
Δρ_max_ = 1.45 e Å^−3^
Δρ_min_ = −0.73 e Å^−3^

### 

Data collection: *APEX2* (Bruker, 2005 ▶); cell refinement: *SAINT* (Bruker, 2005 ▶); data reduction: *SAINT*; program(s) used to solve structure: *SHELXS97* (Sheldrick, 2008 ▶); program(s) used to refine structure: *SHELXL97* (Sheldrick, 2008 ▶); molecular graphics: *SHELXTL* (Sheldrick, 2008 ▶) and *Mercury* (Macrae *et al.*, 2008 ▶); software used to prepare material for publication: *SHELXTL* and *enCIFer* (Allen *et al.*, 2004 ▶).

## Supplementary Material

Crystal structure: contains datablock(s) I, global. DOI: 10.1107/S1600536812031418/jj2145sup1.cif

Structure factors: contains datablock(s) I. DOI: 10.1107/S1600536812031418/jj2145Isup2.hkl

Supplementary material file. DOI: 10.1107/S1600536812031418/jj2145Isup3.cml

Additional supplementary materials:  crystallographic information; 3D view; checkCIF report

## Figures and Tables

**Table 1:** Hydrogen-bond geometry (Å, °)

*D*—H⋯*A*	*D*—H	H⋯*A*	*D*⋯*A*	*D*—H⋯*A*
N4—H4*N*⋯O2^i^	0.85 (2)	2.01 (2)	2.855 (3)	176 (3)
N1—H1*N*⋯O6^ii^	0.87 (2)	2.04 (2)	2.870 (3)	159 (3)

Symmetry codes: (i) 
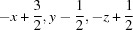
; (ii) 
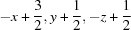
.

**Fig. 1.**
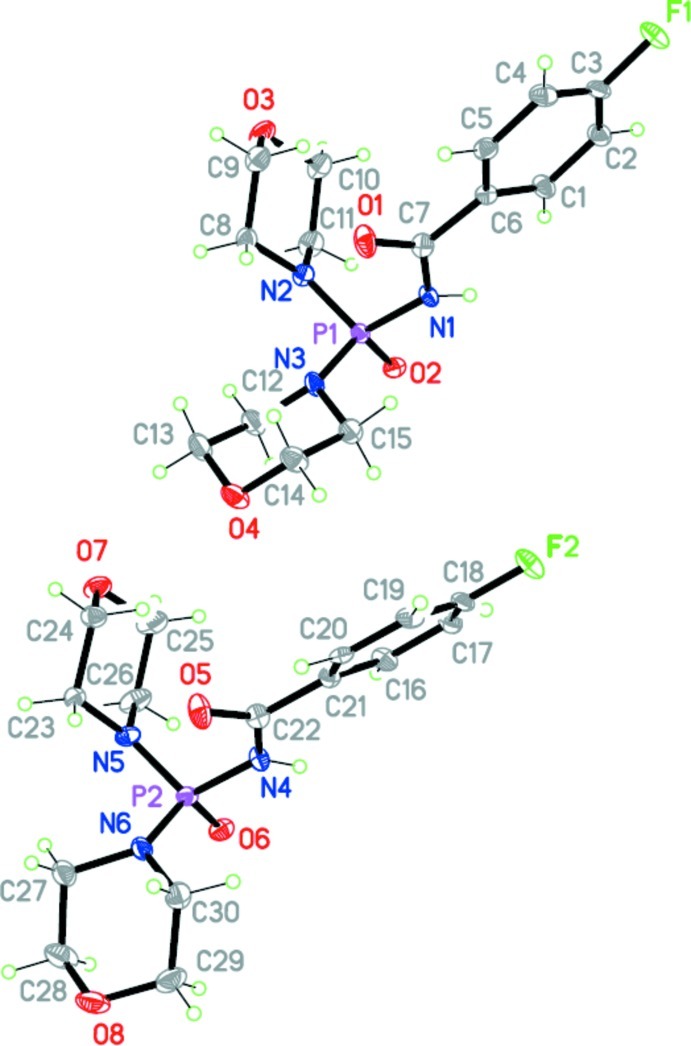
An ORTEP-style plot and atom labeling scheme for the asymmetric unit of the title molecule. Displacement ellipsoids are given at 50% probability level and H atoms are drawn as small spheres of arbitrary radii.

**Fig. 2.**
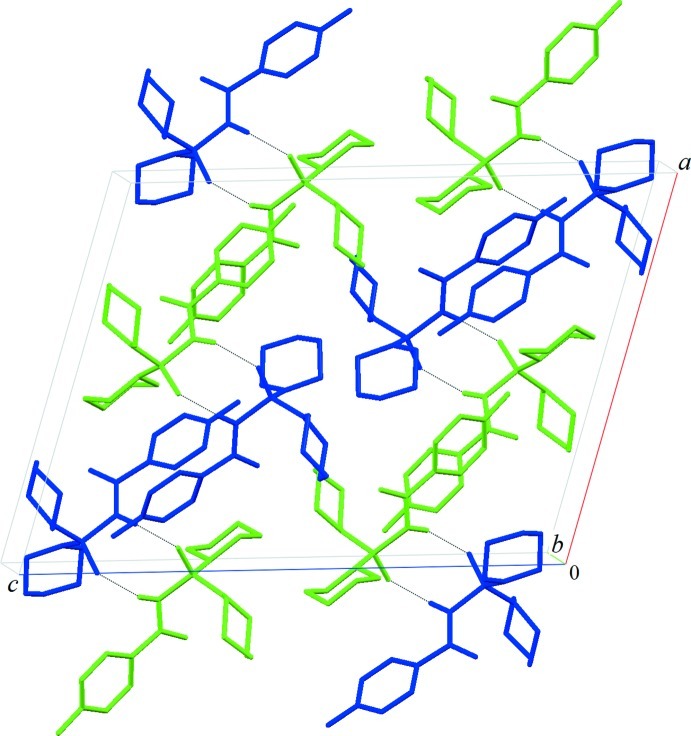
Crystal packing view showing N—H···O(P) hydrogen bond which are shown as dotted lines. The symmetrically independent molecules are shown as different colors and the H atoms not involved in hydrogen bonding have been omitted for the sake of clarity.

## supplementary crystallographic information

### Comment

The structure determination of a monoclinic polymorph of the title molecule, [4-F—C_6_H_4_C(O)NH]P(O)[NC_4_H_8_O]_2_ (Fig. 1), in a *C*2/*c* space group with *Z* = 8 was investigated in ambient temperature, Gholivand *et al.* (2006). Here, we report on a second monoclinic polymorph in a space group *P*2_1_/*n* with *Z* = 8. Cell parameters for both polymorphs exhibit similar dimensions. The *C*2/c structure at 293 K has cell parameters of 15.732 (3), 10.740 (2), 21.553 (4) Å with β of 106.93 (3)° while those of *P*2_1_/*n* at 100 K have smaller cell dimensions indicative of a possible phase change.

The asymmetric unit consists of two independent molecules; in each molecule, the phosphoryl group adopts a *syn* orientation with respect to the N—H unit. In the first monoclinic modification of this compound (Gholivand *et al.*, 2006), the asymmetric unit is composed of one molecule involving disorder in one morpholin-4-yl moiety. The P atom is in a distorted tetrahedral environment as has been noted for other phosphoramides (Pourayoubi, Nečas & Negari, 2012 and Pourayoubi, Tarahhomi *et al.*, 2012).

The P═O, C═O and P—N bond lengths and P—N—C bond angles are within the expected values (Gholivand *et al.*, 2006).

In the crystal, two different intermolecular N—H···O(P) hydrogen bonds make dimeric aggregates. So, each hydrogen-bonded dimer is built of two symmetrically independent molecules (Fig. 2).

### Experimental

4-F—C_6_H_4_C(O)NHP(O)Cl_2_ was prepared according to the literature method reported by Gholivand *et al.* (2006).

To a solution of 4-F—C_6_H_4_C(O)NHP(O)Cl_2_ (0.723 g, 2.825 mmol) in dry acetonitrile (25 ml), a solution of morpholine (0.984 g, 11.300 mmol) in dry acetonitrile (5 ml) was added at 273 K. After 4 h stirring, the solvent was removed and the product was washed with distilled water and the resulting precipitate of 4-F—C_6_H_4_C(O)NHP(O)(NC_4_H_8_O)_2_ was collected. Single crystals were obtained in a try on a reaction between 4-F—C_6_H_4_C(O)NHP(O)(NC_4_H_8_O)_2_ and Sn(CH_3_)_2_Cl_2_ in CH_3_OH under reflux, followed by slow evaporation of the filtered solution at room temperature.

### Refinement

All non-hydrogen atoms were refined anisotropically by full matrix least squares on F^2^. Hydrogen atoms H1N and H4N were found from a Fourier difference map and their N—H distances were fixed at 0.87 (2) Å and were allowed to refine isotropically with 1.20 *U*_eq_ of parent N atoms. All other hydrogen atoms were placed in calculated positions and treated as riding on their parent C atoms with distances C—H = 0.990 Å (CH_2_) and 1.00 Å (CH) with 1.20*U*_eq_ of their parent C atoms.

### Crystal data

**Table:**

C_15_H_21_FN_3_O_4_P	*F*(000) = 1504
*M**_r_* = 357.32	*D*_x_ = 1.413 Mg m^−^^3^
Monoclinic, *P*2_1_/*n*	Mo *K*α radiation, λ = 0.71073 Å
Hall symbol: -P 2yn	Cell parameters from 7455 reflections
*a* = 15.6093 (6) Å	θ = 2.3–26.4°
*b* = 10.7114 (4) Å	µ = 0.20 mm^−^^1^
*c* = 21.0045 (9) Å	*T* = 100 K
β = 106.896 (2)°	Block, colourless
*V* = 3360.3 (2) Å^3^	0.18 × 0.15 × 0.10 mm
*Z* = 8	

### Data collection

**Table:**

Bruker APEXII CCD diffractometer	6912 independent reflections
Radiation source: fine-focus sealed tube	5355 reflections with *I* > 2σ(*I*)
Graphite monochromator	*R*_int_ = 0.037
Detector resolution: 8.33 pixels mm^-1^	θ_max_ = 26.4°, θ_min_ = 1.4°
φ and ω scans	*h* = −19→19
Absorption correction: multi-scan (*SADABS*; Sheldrick, 2004)	*k* = −13→13
*T*_min_ = 0.965, *T*_max_ = 0.980	*l* = −22→26
25230 measured reflections	

### Refinement

**Table:**

Refinement on *F*^2^	Primary atom site location: structure-invariant direct methods
Least-squares matrix: full	Secondary atom site location: difference Fourier map
*R*[*F*^2^ > 2σ(*F*^2^)] = 0.056	Hydrogen site location: inferred from neighbouring sites
*wR*(*F*^2^) = 0.160	H atoms treated by a mixture of independent and constrained refinement
*S* = 1.03	*w* = 1/[σ^2^(*F*_o_^2^) + (0.0816*P*)^2^ + 4.9555*P*] where *P* = (*F*_o_^2^ + 2*F*_c_^2^)/3
6912 reflections	(Δ/σ)_max_ < 0.001
439 parameters	Δρ_max_ = 1.45 e Å^−^^3^
2 restraints	Δρ_min_ = −0.73 e Å^−^^3^

### Special details

**Table:**

Geometry. All e.s.d.'s (except the e.s.d. in the dihedral angle between two l.s. planes) are estimated using the full covariance matrix. The cell e.s.d.'s are taken into account individually in the estimation of e.s.d.'s in distances, angles and torsion angles; correlations between e.s.d.'s in cell parameters are only used when they are defined by crystal symmetry. An approximate (isotropic) treatment of cell e.s.d.'s is used for estimating e.s.d.'s involving l.s. planes.
Refinement. Refinement of *F*^2^ against ALL reflections. The weighted *R*-factor *wR* and goodness of fit *S* are based on *F*^2^, conventional *R*-factors *R* are based on *F*, with *F* set to zero for negative *F*^2^. The threshold expression of *F*^2^ > σ(*F*^2^) is used only for calculating *R*-factors(gt) *etc*. and is not relevant to the choice of reflections for refinement. *R*-factors based on *F*^2^ are statistically about twice as large as those based on *F*, and *R*- factors based on ALL data will be even larger.

### Fractional atomic coordinates and isotropic or equivalent isotropic displacement parameters (Å^2^)

**Table:**

	*x*	*y*	*z*	*U*_iso_*/*U*_eq_	
P1	0.47360 (4)	0.87432 (6)	0.15573 (3)	0.01350 (17)	
P2	0.93848 (4)	0.36293 (6)	0.12376 (3)	0.01408 (17)	
F1	0.07031 (11)	0.82774 (16)	0.31748 (9)	0.0255 (4)	
F2	0.58872 (11)	0.32224 (15)	0.33946 (9)	0.0239 (4)	
O1	0.30622 (14)	0.71437 (19)	0.12959 (10)	0.0250 (5)	
O2	0.54401 (12)	0.95376 (17)	0.19942 (9)	0.0170 (4)	
O3	0.26441 (14)	1.08121 (18)	0.01350 (10)	0.0263 (5)	
O4	0.59087 (13)	0.54123 (18)	0.08540 (10)	0.0238 (5)	
O5	0.76002 (14)	0.22559 (19)	0.10808 (10)	0.0246 (5)	
O6	1.01711 (12)	0.43300 (18)	0.16535 (10)	0.0192 (4)	
O7	0.74106 (13)	0.59113 (17)	−0.00982 (10)	0.0228 (4)	
O8	1.08127 (14)	0.03799 (19)	0.09330 (11)	0.0294 (5)	
N1	0.40849 (14)	0.8319 (2)	0.20362 (11)	0.0149 (5)	
H1N	0.417 (2)	0.872 (3)	0.2410 (11)	0.018*	
N2	0.41816 (15)	0.9499 (2)	0.08931 (11)	0.0165 (5)	
N3	0.50455 (15)	0.7452 (2)	0.12658 (11)	0.0176 (5)	
N4	0.87238 (15)	0.3430 (2)	0.17412 (11)	0.0157 (5)	
H4N	0.8948 (19)	0.374 (3)	0.2123 (10)	0.019*	
N5	0.88937 (14)	0.4404 (2)	0.05651 (11)	0.0164 (5)	
N6	0.95166 (15)	0.2234 (2)	0.09645 (11)	0.0179 (5)	
C1	0.27337 (18)	0.8749 (3)	0.27251 (14)	0.0186 (6)	
H1A	0.3255	0.9257	0.2849	0.022*	
C2	0.20861 (18)	0.8881 (3)	0.30509 (14)	0.0199 (6)	
H2A	0.2152	0.9479	0.3396	0.024*	
C3	0.13450 (17)	0.8125 (2)	0.28632 (14)	0.0172 (6)	
C4	0.12203 (18)	0.7231 (3)	0.23765 (14)	0.0198 (6)	
H4A	0.0707	0.6706	0.2269	0.024*	
C5	0.18645 (17)	0.7119 (2)	0.20477 (14)	0.0173 (5)	
H5A	0.1788	0.6520	0.1702	0.021*	
C6	0.26243 (17)	0.7873 (2)	0.22151 (13)	0.0152 (5)	
C7	0.32636 (18)	0.7737 (2)	0.18122 (13)	0.0168 (5)	
C8	0.35807 (18)	0.8961 (3)	0.02858 (14)	0.0199 (6)	
H8A	0.3567	0.8041	0.0330	0.024*	
H8B	0.3805	0.9159	−0.0098	0.024*	
C9	0.2642 (2)	0.9483 (3)	0.01647 (15)	0.0232 (6)	
H9A	0.2249	0.9147	−0.0259	0.028*	
H9B	0.2397	0.9213	0.0528	0.028*	
C10	0.3181 (2)	1.1302 (3)	0.07509 (15)	0.0242 (6)	
H10A	0.2939	1.1023	0.1114	0.029*	
H10B	0.3160	1.2225	0.0735	0.029*	
C11	0.4140 (2)	1.0871 (2)	0.08929 (15)	0.0226 (6)	
H11A	0.4398	1.1206	0.0550	0.027*	
H11B	0.4498	1.1193	0.1331	0.027*	
C12	0.56485 (19)	0.7619 (3)	0.08400 (15)	0.0225 (6)	
H12A	0.6268	0.7770	0.1122	0.027*	
H12B	0.5454	0.8351	0.0546	0.027*	
C13	0.5620 (2)	0.6472 (3)	0.04299 (15)	0.0249 (6)	
H13A	0.5002	0.6333	0.0141	0.030*	
H13B	0.6015	0.6582	0.0141	0.030*	
C14	0.53270 (19)	0.5219 (3)	0.12520 (15)	0.0226 (6)	
H14A	0.5529	0.4478	0.1538	0.027*	
H14B	0.4715	0.5049	0.0960	0.027*	
C15	0.53018 (19)	0.6333 (2)	0.16835 (14)	0.0204 (6)	
H15A	0.4864	0.6186	0.1935	0.024*	
H15B	0.5898	0.6459	0.2007	0.024*	
C16	0.75142 (17)	0.3982 (2)	0.25373 (14)	0.0173 (5)	
H16A	0.7956	0.4597	0.2545	0.021*	
C17	0.70067 (17)	0.4068 (2)	0.29795 (14)	0.0177 (6)	
H17A	0.7088	0.4741	0.3286	0.021*	
C18	0.63820 (17)	0.3150 (2)	0.29609 (13)	0.0159 (5)	
C19	0.62334 (16)	0.2167 (2)	0.25214 (14)	0.0158 (5)	
H19A	0.5800	0.1546	0.2524	0.019*	
C20	0.67331 (16)	0.2109 (2)	0.20749 (13)	0.0131 (5)	
H20A	0.6630	0.1450	0.1759	0.016*	
C21	0.73824 (17)	0.3000 (2)	0.20819 (13)	0.0142 (5)	
C22	0.78981 (17)	0.2861 (2)	0.15893 (14)	0.0161 (5)	
C23	0.81724 (17)	0.3917 (2)	0.00028 (13)	0.0171 (5)	
H23A	0.8097	0.3012	0.0065	0.021*	
H23B	0.8334	0.4033	−0.0415	0.021*	
C24	0.73018 (18)	0.4588 (2)	−0.00470 (15)	0.0190 (6)	
H24A	0.6832	0.4286	−0.0443	0.023*	
H24B	0.7108	0.4401	0.0352	0.023*	
C25	0.8061 (2)	0.6370 (3)	0.04805 (16)	0.0262 (7)	
H25A	0.7871	0.6179	0.0880	0.031*	
H25B	0.8112	0.7288	0.0449	0.031*	
C26	0.89630 (19)	0.5771 (2)	0.05444 (16)	0.0244 (6)	
H26A	0.9174	0.6018	0.0161	0.029*	
H26B	0.9406	0.6070	0.0956	0.029*	
C27	0.98143 (19)	0.2023 (3)	0.03698 (15)	0.0229 (6)	
H27A	0.9799	0.2821	0.0129	0.027*	
H27B	0.9401	0.1432	0.0068	0.027*	
C28	1.0750 (2)	0.1502 (3)	0.05604 (18)	0.0305 (7)	
H28A	1.0927	0.1334	0.0153	0.037*	
H28B	1.1169	0.2127	0.0828	0.037*	
C29	1.0563 (2)	0.0604 (3)	0.15248 (16)	0.0280 (7)	
H29A	1.0983	0.1214	0.1806	0.034*	
H29B	1.0610	−0.0184	0.1779	0.034*	
C30	0.9619 (2)	0.1102 (3)	0.13704 (15)	0.0221 (6)	
H30A	0.9191	0.0459	0.1129	0.027*	
H30B	0.9479	0.1291	0.1791	0.027*	

### Atomic displacement parameters (Å^2^)

**Table:**

	*U*^11^	*U*^22^	*U*^33^	*U*^12^	*U*^13^	*U*^23^
P1	0.0156 (3)	0.0121 (3)	0.0144 (3)	0.0020 (2)	0.0069 (3)	0.0010 (3)
P2	0.0130 (3)	0.0138 (3)	0.0165 (4)	0.0014 (2)	0.0060 (3)	0.0030 (3)
F1	0.0196 (8)	0.0309 (9)	0.0323 (10)	0.0007 (7)	0.0173 (7)	−0.0010 (8)
F2	0.0236 (8)	0.0232 (8)	0.0321 (10)	−0.0011 (7)	0.0194 (7)	−0.0030 (7)
O1	0.0283 (11)	0.0296 (11)	0.0193 (11)	−0.0100 (9)	0.0104 (9)	−0.0112 (9)
O2	0.0146 (9)	0.0176 (9)	0.0180 (10)	0.0000 (7)	0.0034 (7)	0.0024 (8)
O3	0.0328 (11)	0.0195 (10)	0.0199 (11)	0.0081 (9)	−0.0026 (9)	−0.0024 (8)
O4	0.0260 (11)	0.0196 (10)	0.0305 (12)	0.0071 (8)	0.0155 (9)	0.0017 (9)
O5	0.0287 (11)	0.0272 (11)	0.0211 (11)	−0.0105 (9)	0.0124 (9)	−0.0097 (9)
O6	0.0142 (9)	0.0230 (10)	0.0201 (10)	−0.0004 (8)	0.0045 (8)	0.0065 (8)
O7	0.0235 (10)	0.0166 (9)	0.0240 (11)	0.0052 (8)	0.0002 (8)	0.0008 (8)
O8	0.0281 (11)	0.0238 (11)	0.0389 (13)	0.0130 (9)	0.0140 (10)	0.0058 (10)
N1	0.0151 (11)	0.0159 (10)	0.0157 (11)	−0.0010 (9)	0.0077 (9)	−0.0041 (9)
N2	0.0214 (11)	0.0114 (10)	0.0158 (11)	0.0024 (9)	0.0041 (9)	0.0001 (9)
N3	0.0260 (12)	0.0144 (11)	0.0162 (12)	0.0057 (9)	0.0120 (10)	0.0037 (9)
N4	0.0168 (11)	0.0171 (11)	0.0151 (11)	−0.0028 (9)	0.0075 (9)	−0.0033 (9)
N5	0.0145 (11)	0.0133 (10)	0.0198 (12)	0.0004 (8)	0.0026 (9)	0.0028 (9)
N6	0.0235 (12)	0.0165 (11)	0.0163 (11)	0.0052 (9)	0.0102 (9)	0.0030 (9)
C1	0.0153 (13)	0.0222 (14)	0.0196 (14)	−0.0039 (10)	0.0073 (11)	−0.0049 (11)
C2	0.0168 (13)	0.0239 (14)	0.0191 (14)	0.0007 (11)	0.0054 (11)	−0.0060 (11)
C3	0.0121 (12)	0.0205 (13)	0.0211 (14)	0.0046 (10)	0.0081 (11)	0.0060 (11)
C4	0.0133 (12)	0.0200 (13)	0.0259 (15)	−0.0025 (10)	0.0055 (11)	0.0016 (11)
C5	0.0154 (13)	0.0177 (13)	0.0169 (13)	−0.0003 (10)	0.0018 (10)	−0.0017 (11)
C6	0.0135 (12)	0.0150 (12)	0.0164 (13)	0.0011 (10)	0.0036 (10)	0.0008 (10)
C7	0.0205 (13)	0.0134 (12)	0.0168 (14)	−0.0010 (10)	0.0057 (11)	−0.0006 (10)
C8	0.0252 (14)	0.0183 (13)	0.0162 (14)	0.0028 (11)	0.0061 (11)	−0.0019 (11)
C9	0.0289 (15)	0.0178 (13)	0.0202 (15)	0.0035 (11)	0.0026 (12)	−0.0035 (11)
C10	0.0327 (16)	0.0163 (13)	0.0193 (15)	0.0074 (12)	0.0008 (12)	−0.0024 (11)
C11	0.0305 (16)	0.0130 (13)	0.0228 (15)	0.0008 (11)	0.0055 (12)	0.0020 (11)
C12	0.0262 (15)	0.0214 (14)	0.0241 (15)	0.0034 (12)	0.0140 (12)	0.0021 (12)
C13	0.0324 (16)	0.0238 (15)	0.0238 (16)	0.0070 (12)	0.0165 (13)	0.0015 (12)
C14	0.0240 (14)	0.0189 (13)	0.0280 (16)	0.0028 (11)	0.0122 (12)	0.0024 (12)
C15	0.0251 (14)	0.0175 (13)	0.0208 (14)	0.0038 (11)	0.0101 (12)	0.0047 (11)
C16	0.0127 (12)	0.0171 (13)	0.0229 (14)	−0.0039 (10)	0.0064 (10)	−0.0043 (11)
C17	0.0156 (13)	0.0187 (13)	0.0183 (14)	−0.0011 (10)	0.0042 (11)	−0.0068 (11)
C18	0.0124 (12)	0.0186 (13)	0.0190 (14)	0.0043 (10)	0.0084 (10)	0.0027 (11)
C19	0.0113 (12)	0.0129 (12)	0.0228 (14)	−0.0009 (9)	0.0043 (10)	0.0016 (10)
C20	0.0095 (11)	0.0120 (11)	0.0155 (13)	0.0020 (9)	−0.0002 (10)	0.0014 (10)
C21	0.0145 (12)	0.0131 (12)	0.0151 (13)	0.0003 (9)	0.0045 (10)	0.0017 (10)
C22	0.0180 (13)	0.0122 (12)	0.0187 (14)	−0.0033 (10)	0.0061 (11)	−0.0004 (10)
C23	0.0183 (13)	0.0167 (12)	0.0156 (13)	0.0021 (10)	0.0036 (11)	−0.0009 (10)
C24	0.0179 (13)	0.0159 (13)	0.0221 (14)	0.0012 (10)	0.0041 (11)	−0.0016 (11)
C25	0.0281 (15)	0.0127 (13)	0.0316 (17)	0.0043 (11)	−0.0010 (13)	−0.0011 (12)
C26	0.0226 (14)	0.0143 (13)	0.0307 (16)	−0.0031 (11)	−0.0011 (12)	0.0069 (12)
C27	0.0268 (15)	0.0242 (14)	0.0215 (15)	0.0043 (12)	0.0130 (12)	0.0026 (12)
C28	0.0291 (16)	0.0278 (16)	0.0419 (19)	0.0094 (13)	0.0221 (15)	0.0092 (14)
C29	0.0309 (16)	0.0234 (15)	0.0260 (16)	0.0081 (12)	0.0022 (13)	0.0091 (13)
C30	0.0296 (15)	0.0168 (13)	0.0210 (15)	0.0021 (11)	0.0091 (12)	0.0034 (11)

### Geometric parameters (Å, º)

**Table:**

P1—O2	1.4791 (19)	C9—H9B	0.9900
P1—N2	1.628 (2)	C10—C11	1.511 (4)
P1—N3	1.641 (2)	C10—H10A	0.9900
P1—N1	1.687 (2)	C10—H10B	0.9900
P2—O6	1.4866 (19)	C11—H11A	0.9900
P2—N5	1.626 (2)	C11—H11B	0.9900
P2—N6	1.635 (2)	C12—C13	1.495 (4)
P2—N4	1.692 (2)	C12—H12A	0.9900
F1—C3	1.356 (3)	C12—H12B	0.9900
F2—C18	1.357 (3)	C13—H13A	0.9900
O1—C7	1.217 (3)	C13—H13B	0.9900
O3—C10	1.422 (3)	C14—C15	1.505 (4)
O3—C9	1.425 (3)	C14—H14A	0.9900
O4—C14	1.417 (3)	C14—H14B	0.9900
O4—C13	1.432 (3)	C15—H15A	0.9900
O5—C22	1.220 (3)	C15—H15B	0.9900
O7—C25	1.426 (3)	C16—C17	1.388 (4)
O7—C24	1.435 (3)	C16—C21	1.396 (4)
O8—C28	1.422 (4)	C16—H16A	0.9500
O8—C29	1.427 (4)	C17—C18	1.378 (4)
N1—C7	1.380 (3)	C17—H17A	0.9500
N1—H1N	0.869 (17)	C18—C19	1.375 (4)
N2—C8	1.464 (3)	C19—C20	1.385 (4)
N2—C11	1.471 (3)	C19—H19A	0.9500
N3—C15	1.471 (3)	C20—C21	1.389 (3)
N3—C12	1.486 (3)	C20—H20A	0.9500
N4—C22	1.377 (3)	C21—C22	1.491 (4)
N4—H4N	0.846 (18)	C23—C24	1.514 (4)
N5—C26	1.470 (3)	C23—H23A	0.9900
N5—C23	1.470 (3)	C23—H23B	0.9900
N6—C30	1.465 (3)	C24—H24A	0.9900
N6—C27	1.471 (3)	C24—H24B	0.9900
C1—C2	1.383 (4)	C25—C26	1.517 (4)
C1—C6	1.397 (4)	C25—H25A	0.9900
C1—H1A	0.9500	C25—H25B	0.9900
C2—C3	1.373 (4)	C26—H26A	0.9900
C2—H2A	0.9500	C26—H26B	0.9900
C3—C4	1.373 (4)	C27—C28	1.504 (4)
C4—C5	1.381 (4)	C27—H27A	0.9900
C4—H4A	0.9500	C27—H27B	0.9900
C5—C6	1.392 (4)	C28—H28A	0.9900
C5—H5A	0.9500	C28—H28B	0.9900
C6—C7	1.491 (4)	C29—C30	1.512 (4)
C8—C9	1.519 (4)	C29—H29A	0.9900
C8—H8A	0.9900	C29—H29B	0.9900
C8—H8B	0.9900	C30—H30A	0.9900
C9—H9A	0.9900	C30—H30B	0.9900
			
O2—P1—N2	111.08 (11)	O4—C13—H13A	109.7
O2—P1—N3	118.04 (11)	C12—C13—H13A	109.7
N2—P1—N3	103.92 (11)	O4—C13—H13B	109.7
O2—P1—N1	104.63 (11)	C12—C13—H13B	109.7
N2—P1—N1	112.50 (12)	H13A—C13—H13B	108.2
N3—P1—N1	106.82 (11)	O4—C14—C15	111.9 (2)
O6—P2—N5	110.68 (11)	O4—C14—H14A	109.2
O6—P2—N6	120.07 (12)	C15—C14—H14A	109.2
N5—P2—N6	103.74 (12)	O4—C14—H14B	109.2
O6—P2—N4	104.22 (11)	C15—C14—H14B	109.2
N5—P2—N4	112.86 (12)	H14A—C14—H14B	107.9
N6—P2—N4	105.41 (11)	N3—C15—C14	109.7 (2)
C10—O3—C9	109.7 (2)	N3—C15—H15A	109.7
C14—O4—C13	110.1 (2)	C14—C15—H15A	109.7
C25—O7—C24	110.3 (2)	N3—C15—H15B	109.7
C28—O8—C29	110.2 (2)	C14—C15—H15B	109.7
C7—N1—P1	125.42 (19)	H15A—C15—H15B	108.2
C7—N1—H1N	115 (2)	C17—C16—C21	120.6 (2)
P1—N1—H1N	116 (2)	C17—C16—H16A	119.7
C8—N2—C11	112.1 (2)	C21—C16—H16A	119.7
C8—N2—P1	126.66 (18)	C18—C17—C16	118.1 (2)
C11—N2—P1	120.65 (19)	C18—C17—H17A	121.0
C15—N3—C12	110.3 (2)	C16—C17—H17A	121.0
C15—N3—P1	121.53 (18)	F2—C18—C19	118.5 (2)
C12—N3—P1	115.43 (18)	F2—C18—C17	118.5 (2)
C22—N4—P2	127.66 (19)	C19—C18—C17	123.1 (2)
C22—N4—H4N	120 (2)	C18—C19—C20	118.1 (2)
P2—N4—H4N	113 (2)	C18—C19—H19A	121.0
C26—N5—C23	112.1 (2)	C20—C19—H19A	120.9
C26—N5—P2	120.94 (19)	C19—C20—C21	121.0 (2)
C23—N5—P2	125.07 (18)	C19—C20—H20A	119.5
C30—N6—C27	111.0 (2)	C21—C20—H20A	119.5
C30—N6—P2	123.80 (19)	C20—C21—C16	119.1 (2)
C27—N6—P2	122.78 (19)	C20—C21—C22	117.6 (2)
C2—C1—C6	120.3 (2)	C16—C21—C22	123.3 (2)
C2—C1—H1A	119.9	O5—C22—N4	122.1 (2)
C6—C1—H1A	119.9	O5—C22—C21	121.0 (2)
C3—C2—C1	118.3 (3)	N4—C22—C21	116.9 (2)
C3—C2—H2A	120.9	N5—C23—C24	110.5 (2)
C1—C2—H2A	120.9	N5—C23—H23A	109.6
F1—C3—C2	118.0 (2)	C24—C23—H23A	109.6
F1—C3—C4	118.6 (2)	N5—C23—H23B	109.6
C2—C3—C4	123.4 (2)	C24—C23—H23B	109.6
C3—C4—C5	117.8 (2)	H23A—C23—H23B	108.1
C3—C4—H4A	121.1	O7—C24—C23	110.5 (2)
C5—C4—H4A	121.1	O7—C24—H24A	109.5
C4—C5—C6	120.9 (3)	C23—C24—H24A	109.5
C4—C5—H5A	119.5	O7—C24—H24B	109.5
C6—C5—H5A	119.5	C23—C24—H24B	109.5
C5—C6—C1	119.2 (2)	H24A—C24—H24B	108.1
C5—C6—C7	117.5 (2)	O7—C25—C26	110.0 (2)
C1—C6—C7	123.2 (2)	O7—C25—H25A	109.7
O1—C7—N1	120.9 (2)	C26—C25—H25A	109.7
O1—C7—C6	121.0 (2)	O7—C25—H25B	109.7
N1—C7—C6	118.1 (2)	C26—C25—H25B	109.7
N2—C8—C9	110.1 (2)	H25A—C25—H25B	108.2
N2—C8—H8A	109.6	N5—C26—C25	110.5 (2)
C9—C8—H8A	109.6	N5—C26—H26A	109.6
N2—C8—H8B	109.6	C25—C26—H26A	109.6
C9—C8—H8B	109.6	N5—C26—H26B	109.6
H8A—C8—H8B	108.2	C25—C26—H26B	109.6
O3—C9—C8	111.1 (2)	H26A—C26—H26B	108.1
O3—C9—H9A	109.4	N6—C27—C28	110.7 (2)
C8—C9—H9A	109.4	N6—C27—H27A	109.5
O3—C9—H9B	109.4	C28—C27—H27A	109.5
C8—C9—H9B	109.4	N6—C27—H27B	109.5
H9A—C9—H9B	108.0	C28—C27—H27B	109.5
O3—C10—C11	110.8 (2)	H27A—C27—H27B	108.1
O3—C10—H10A	109.5	O8—C28—C27	111.5 (2)
C11—C10—H10A	109.5	O8—C28—H28A	109.3
O3—C10—H10B	109.5	C27—C28—H28A	109.3
C11—C10—H10B	109.5	O8—C28—H28B	109.3
H10A—C10—H10B	108.1	C27—C28—H28B	109.3
N2—C11—C10	110.3 (2)	H28A—C28—H28B	108.0
N2—C11—H11A	109.6	O8—C29—C30	111.6 (2)
C10—C11—H11A	109.6	O8—C29—H29A	109.3
N2—C11—H11B	109.6	C30—C29—H29A	109.3
C10—C11—H11B	109.6	O8—C29—H29B	109.3
H11A—C11—H11B	108.1	C30—C29—H29B	109.3
N3—C12—C13	109.3 (2)	H29A—C29—H29B	108.0
N3—C12—H12A	109.8	N6—C30—C29	110.6 (2)
C13—C12—H12A	109.8	N6—C30—H30A	109.5
N3—C12—H12B	109.8	C29—C30—H30A	109.5
C13—C12—H12B	109.8	N6—C30—H30B	109.5
H12A—C12—H12B	108.3	C29—C30—H30B	109.5
O4—C13—C12	110.0 (2)	H30A—C30—H30B	108.1
			
O2—P1—N1—C7	169.2 (2)	P1—N2—C8—C9	119.3 (2)
N2—P1—N1—C7	48.5 (2)	C10—O3—C9—C8	−61.2 (3)
N3—P1—N1—C7	−64.9 (2)	N2—C8—C9—O3	56.1 (3)
O2—P1—N2—C8	165.5 (2)	C9—O3—C10—C11	61.6 (3)
N3—P1—N2—C8	37.6 (2)	C8—N2—C11—C10	52.5 (3)
N1—P1—N2—C8	−77.6 (2)	P1—N2—C11—C10	−119.2 (2)
O2—P1—N2—C11	−24.1 (2)	O3—C10—C11—N2	−57.0 (3)
N3—P1—N2—C11	−152.0 (2)	C15—N3—C12—C13	56.9 (3)
N1—P1—N2—C11	92.9 (2)	P1—N3—C12—C13	−160.6 (2)
O2—P1—N3—C15	76.8 (2)	C14—O4—C13—C12	61.5 (3)
N2—P1—N3—C15	−159.7 (2)	N3—C12—C13—O4	−60.0 (3)
N1—P1—N3—C15	−40.6 (2)	C13—O4—C14—C15	−59.7 (3)
O2—P1—N3—C12	−61.2 (2)	C12—N3—C15—C14	−54.3 (3)
N2—P1—N3—C12	62.3 (2)	P1—N3—C15—C14	165.87 (19)
N1—P1—N3—C12	−178.56 (19)	O4—C14—C15—N3	56.1 (3)
O6—P2—N4—C22	178.2 (2)	C21—C16—C17—C18	0.9 (4)
N5—P2—N4—C22	58.0 (3)	C16—C17—C18—F2	179.5 (2)
N6—P2—N4—C22	−54.5 (2)	C16—C17—C18—C19	−0.8 (4)
O6—P2—N5—C26	−25.8 (2)	F2—C18—C19—C20	179.2 (2)
N6—P2—N5—C26	−155.8 (2)	C17—C18—C19—C20	−0.5 (4)
N4—P2—N5—C26	90.6 (2)	C18—C19—C20—C21	1.6 (4)
O6—P2—N5—C23	171.3 (2)	C19—C20—C21—C16	−1.5 (4)
N6—P2—N5—C23	41.3 (2)	C19—C20—C21—C22	179.3 (2)
N4—P2—N5—C23	−72.3 (2)	C17—C16—C21—C20	0.2 (4)
O6—P2—N6—C30	79.2 (2)	C17—C16—C21—C22	179.3 (2)
N5—P2—N6—C30	−156.6 (2)	P2—N4—C22—O5	11.3 (4)
N4—P2—N6—C30	−37.8 (2)	P2—N4—C22—C21	−169.64 (19)
O6—P2—N6—C27	−81.7 (2)	C20—C21—C22—O5	22.0 (4)
N5—P2—N6—C27	42.5 (2)	C16—C21—C22—O5	−157.1 (3)
N4—P2—N6—C27	161.3 (2)	C20—C21—C22—N4	−157.1 (2)
C6—C1—C2—C3	0.5 (4)	C16—C21—C22—N4	23.8 (4)
C1—C2—C3—F1	−178.6 (2)	C26—N5—C23—C24	−52.1 (3)
C1—C2—C3—C4	1.1 (4)	P2—N5—C23—C24	112.1 (2)
F1—C3—C4—C5	177.7 (2)	C25—O7—C24—C23	−61.5 (3)
C2—C3—C4—C5	−2.0 (4)	N5—C23—C24—O7	55.9 (3)
C3—C4—C5—C6	1.3 (4)	C24—O7—C25—C26	61.8 (3)
C4—C5—C6—C1	0.2 (4)	C23—N5—C26—C25	52.7 (3)
C4—C5—C6—C7	−176.9 (2)	P2—N5—C26—C25	−112.2 (2)
C2—C1—C6—C5	−1.2 (4)	O7—C25—C26—N5	−57.1 (3)
C2—C1—C6—C7	175.8 (3)	C30—N6—C27—C28	−53.3 (3)
P1—N1—C7—O1	22.8 (4)	P2—N6—C27—C28	109.8 (3)
P1—N1—C7—C6	−156.19 (19)	C29—O8—C28—C27	−59.2 (3)
C5—C6—C7—O1	11.5 (4)	N6—C27—C28—O8	56.7 (3)
C1—C6—C7—O1	−165.6 (3)	C28—O8—C29—C30	58.9 (3)
C5—C6—C7—N1	−169.6 (2)	C27—N6—C30—C29	52.9 (3)
C1—C6—C7—N1	13.4 (4)	P2—N6—C30—C29	−110.0 (3)
C11—N2—C8—C9	−51.9 (3)	O8—C29—C30—N6	−56.0 (3)

### Hydrogen-bond geometry (Å, º)

**Table:**

*D*—H···*A*	*D*—H	H···*A*	*D*···*A*	*D*—H···*A*
N4—H4*N*···O2^i^	0.85 (2)	2.01 (2)	2.855 (3)	176 (3)
N1—H1*N*···O6^ii^	0.87 (2)	2.04 (2)	2.870 (3)	159 (3)

Symmetry codes: (i) −*x*+3/2, *y*−1/2, −*z*+1/2; (ii) −*x*+3/2, *y*+1/2, −*z*+1/2.
